# Supplementary material for: The evolution of convex trade-offs enables the transition towards multicellularity
Source: Nat Commun. 2021 Jul 9;12:4222. doi: 10.1038/s41467-021-24503-z (PMC8270964; doi:10.1038/s41467-021-24503-z)
Supplement: Supplementary file 1 — Supplementary Information [file 41467_2021_24503_MOESM1_ESM.pdf]

Supplementary information

The evolution of convex trade-offs enables the transition towards multicellularity

Joana P. Bernardes, Uwe John, Noemi Woltermann, Martha Valiadi, Ruben J. Hermann, Lutz Becks

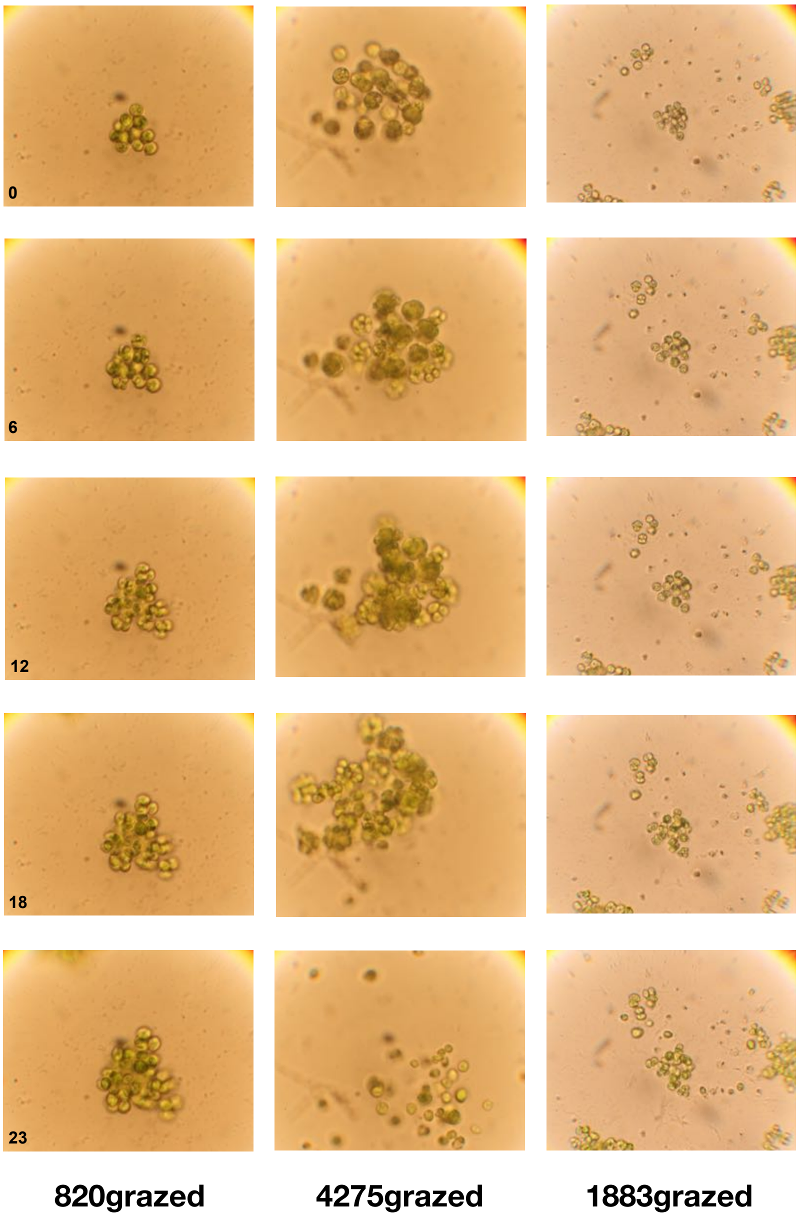

Figure S1: Representative microscopic images of growth of cell groups over 23hrs. Left: isolate from selection line started with cc-850, middle: cc-4275, right: cc-1883. Algal cells were added to a growth chamber of an 8-well chambered cover glass filled with COOL160 medium. Images were taken with an inverse microscope (magnification 200x) and a Nikon camera attached to it. Images were taken every 5 minutes for 24 hours.

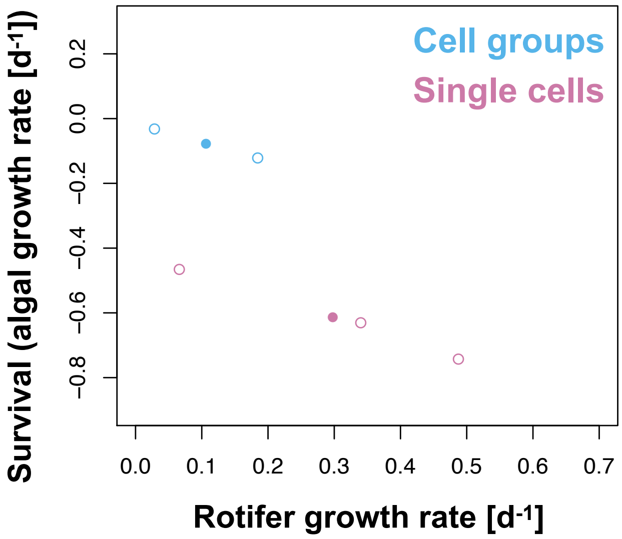

Figure S2: Correlation between algal survival and defense measured as algal and rotifer growth rate in chemostats for two different algal isolates either growing in cell groups or single cells. Chemostats were inoculated with isogenic lines of single cells or cell groups. Algal populations grew to steady state before rotifers were added. Algal and rotifer growth rates were estimated as the average growth rate from day when rotifers had positive growth rates till the day of the rotifer maximum population size. Open symbols replicates (cell groups: 2; single cells: 3), filled symbols represent the average. Source data are provided as a Source Data file.

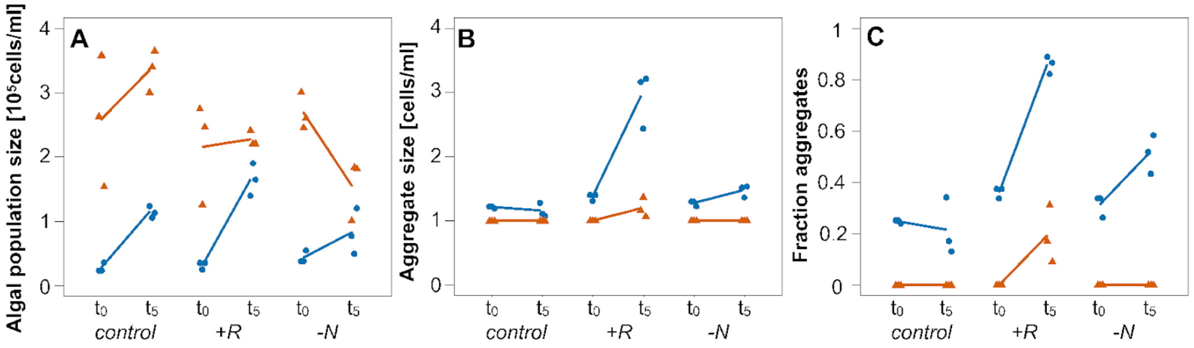

Figure S3: Phenotypic response of isolated algal clones that have previous been selected by competition (red) or predation (blue) to the presence of rotifers (+R) and reduced nitrogen (-N). A) Differences in the cell densities before the addition of a stressor (T_0) and 5 days later T_5). B) Difference in aggregate size and C) fraction of aggregates in the algal population. Shown are replicates (points) and the mean of three replicates (line). Source data are provided as a Source Data file.

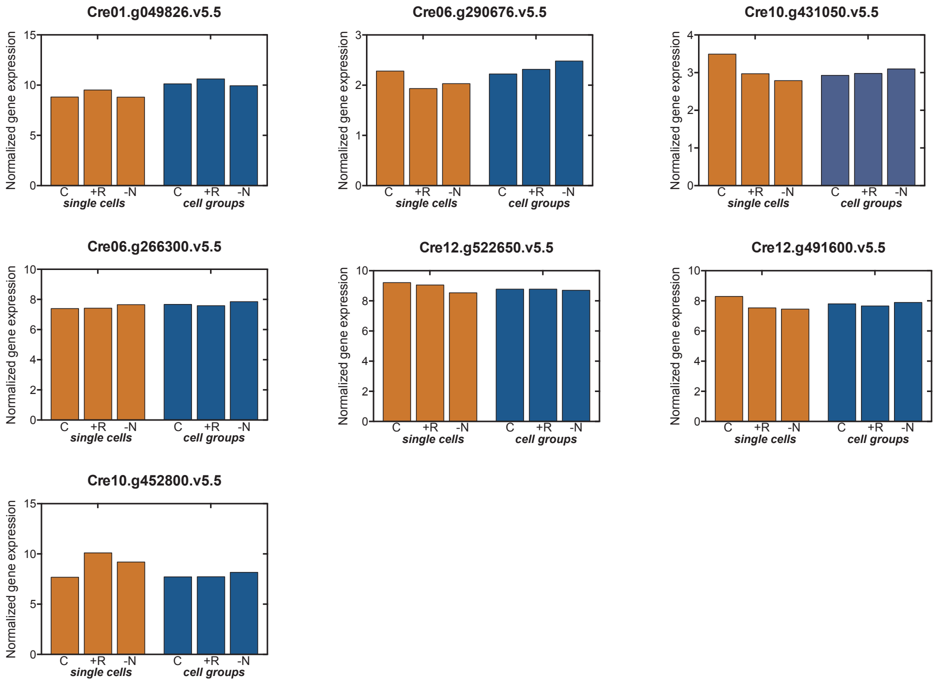

Figure S4: Expression profiles of the five shared variants with amino acid changes. Normalized expression extracted from the transcription profiles of the algal. *Single cells* selection regiment expression depicted in orange and *cell groups* in blue bars, the individual bars represent the treatments the clones were exposed to.

Table S1: Reference strain mutations. List of *Chlamydomonas rheinhardtii* strains used to set up the selection experiment, specific mutations and mating type, and phenotype described in prevous studies. All strains were purchased from the Chlamydomonas strain collection at Chlamydomonas Resource Center. Mutation refers to the culture collections description.

**Strain**	**Mutation**	**Phenotype**
cc850	*cw18 mt-*	*Wall deficient for cell clusters*
cc1009	*mt-*	*Wild type*
cc1144	*sr1 msr1 nr1 act2 pyr1 NIT+ mt-*	*Antibiotic resistance and metabolic inhibitors*
cc1883	*cw15 NIT+ mt-*	*Wall deficient mutant*
cc2531	*vfl2 mt-*	*Variable number of flagella*
cc2698	*cia2*	*Altered CO_2 assimilation*
cc2854	*pf18 cw92 mt-*	*Wall deficient and motility impairment*
cc3056	*cw15 chIB (disruption) mt-*	*Chlorophyll deficient and wall deficient*
cc3754	*fa2-4 mt-*	*Impaired flagellar autonomy*
cc4275	*ars11 (snrk2.1) mt-*	*Impaired response to sulfur starvation*

Table S2: Assembly and variant information of sequenced *C. reinhardtii* strains

Strain and environment	Morphology*	ID	assembled bp	Contigs (#)	average length (bp)	N50	Variants (#)
4275ungrazed	*S1*	S1	98983276	182064	544	1587	101272
4275grazed	*C2*	S2	104345484	194143	537	1522	109792
1144ungrazed	*S1*	S3	100798433	180803	558	1889	283096
1144grazed	*C2*	S4	95910447	196255	489	1274	129514
1009ungrazed	*S1*	S5	108241743	190832	567	1738	230465
1009grazed	*C2*	S6	97706888	174816	559	1679	235437
850ungrazed	*S2*	S7	96431227	174952	551	1626	233848
850grazed	*C2*	S8	108651670	195158	556	1725	164350
3754ungrazed	*S2*	S9	109029565	206358	528	1356	134476
3754grazed	*C1*	S10	96017187	197415	486	1143	97955
3056ungrazed	*S2*	S11	99075774	197652	501	1283	126676
3056grazed	*C2*	S12	103694500	198328	523	1235	212232
2698ungrazed	*S2*	S13	113159208	230200	492	1962	203608
2698grazed	*C2*	S14	93766290	221712	423	680	139844
1883ungrazed	*S2*	S15	101794564	204711	497	1183	66673
1883grazed	*C1*	S16	108239427	232747	465	967	179801
2854ungrazed	*S2*	S17	96155163	196467	489	1173	31731
2854grazed	*C2*	S18	96730316	240605	402	521	119892

* *S1*: single cells with no-motile single cells. *S2*: single cells with some motile single cells present; *S3*: single cells with majority motile single cells; *C1*: cell groups with no single cells *C2:* cell groups with non-motile single cells; *C3:* cell groups with motile single cells.
