# Supplementary material for: The evolution of convex trade-offs enables the transition towards multicellularity
Source: Nat Commun. 2021 Jul 9;12:4222. doi: 10.1038/s41467-021-24503-z (PMC8270964; doi:10.1038/s41467-021-24503-z)
Supplement: Supplementary file 3 — Description of Additional Supplementary Data [file 41467_2021_24503_MOESM3_ESM.docx]

Headings Supplementary Data.

**Supplementary Data 1:** List of differentially expressed genes in Herron et al.^1^ and the present study.

**Supplementary Data 2:** Shared variants within morphotype groups from genome analyses (single cells, cell groups).
